# The 2nd meeting of the Campania Society of Oncology Immunotherapy (SCITO): focus on hepatocellular carcinoma, kidney and bladder cancer

**DOI:** 10.1186/s40425-015-0105-x

**Published:** 2016-01-19

**Authors:** Bruno Daniele, Bruno Sangro, Daniel Petrylak, Fabio Calabrò, Giacomo Cartenì, Vincenzo Montesarchio, Sabino De Placido, Paolo A. Ascierto

**Affiliations:** Department of Oncology, A.O. “G. Rummo”, Benevento, Italy; Liver Unit, Clinica Universidad de Navarra, IDISNA and CIBEREHD, Pamplona, Spain; Genitourinary Oncology Research Program, Yale Comprehensive Cancer Center, Yale School of Medicine, New Haven, CT USA; San Camillo-Forlanini Hospital, Rome, Italy; Unit of Medical Oncology, Dipartimento di Oncopneumoematologia, A.O.R.N. “A. Cardarelli”, Naples, Italy; Unit of Oncology, A.O.R.N. dei COLLI “Ospedali Monaldi-Cotugno-CTO”, Naples, Italy; Department of Molecular and Clinical Endocrinology and Oncology, University “Federico II”, Naples, Italy; Unit of Melanoma, Cancer Immunotherapy and Innovative Therapies, Istituto Nazionale Tumori Fondazione “G. Pascale”, Via Mariano Semmola, 80131 Naples, Italy

**Keywords:** Immunotherapy, Checkpoint inhibitors, Cell-based vaccines, Peptide vaccines, Solid tumors

## Abstract

In this paper, we review recent advances in immuno-oncology with particular focus on liver, kidney, and bladder cancers as discussed at the 2nd meeting of the Campania Society of Oncology Immunotherapy (SCITO).

## Introduction

The immune system has a role in both protecting against tumor development and promoting tumor growth in cancer with these contrasting roles together referred to as immunoediting [1]. This process involves three defined phases, beginning with the immunosurveillance (elimination) phase which is characterized by antigen presentation and T cell activation and the destruction of nascent tumor cells and control of tumor growth. The equilibrium phase involves a steady-state between tumor growth enhancement and inhibition. Finally, in the escape phase, cancer progression is favored by the outgrowth of tumor cells that can suppress or escape the immune system.

Immunity can be divided into innate and adaptive responses. Innate immunity refers to myeloid and lymphoid cells that exert a rapid effector function, while adaptive immunity is driven by T- and B-lymphocytes that express antigen receptors produced by site-specific somatic recombination. Adoptive immunity has greater specificity than innate in retaining antigen memory. The T-cell response is regulated by a balance of activating and inhibitory signals, with checkpoints to limit an ongoing immune response and prevent damage to healthy tissues. Programmed cell death protein 1 (PD-1) and cytotoxic T-lymphocyte-associated protein 4 (CTLA-4) are two examples of inhibitory checkpoints. T-regulatory (Treg) cells are crucially involved in the immune response to cancer and tumor-infiltrating T-lymphocyte cell (TIL) density has a positive prognostic association with overall survival (OS) of patients with various cancer types, including advanced ovarian carcinoma, non-small cell lung (NSCLC), colorectal, breast, head and neck, and kidney cancer as well as melanoma [2]. Conversely, Treg infiltration has been reported to predict a poorer outcome in early-stage NSCLC and renal cell carcinoma.

The approval of sipuleucel-T for the treatment of prostate cancer was the first immuno-oncology therapy to be approved. This was followed by the approval of ipilimumab, an antibody to CTLA-4, for the treatment of metastatic melanoma in 2011. The anti PD-1 antibodies nivolumab (in melanoma and subsequently NSCLC) and pembrolizumab in melanoma have also since been approved, and a large number of immunotherapies are being investigated for their potential activity across many different types of cancer.

Targeting checkpoint and activating pathways is an evolving approach to cancer therapy and their clinical use has highlighted a number of important considerations (Figure). Firstly, immunotherapy agents may be associated with patterns of response that differ from those seen with other treatment modalities (i.e. chemotherapy) and so their use may require the development of different response criteria [3]. For example, a response to CTLA-4 or PD-1 checkpoint blockade may be observed after an initial increase in tumor volume caused by the infiltration of tumor masses through stimulation of immunocompetent cells. In addition, a reduction in tumor burden may be observed after the appearance of new lesions. These observations suggest that planned treatment should be continued regardless of an increase in volume of existing lesions or the early appearance of new lesions. Moreover, durable responses may be observed even after cessation of treatment, suggesting an ability of immunotherapy to reset the equilibrium between host and tumor [4]. Current RECIST and WHO criteria might not be appropriate to asses these types of responses and there is a need for new immune-related response criteria to evaluate these treatments.

Another consideration is that immunotherapies such as anti-CTLA-4 and anti-PD1 antibodies can act regardless of patient characteristics (e.g. age, gender, performance status, prior therapies) and tumor characteristics (e.g. histology, mutation status) [5, 6].

Defining predictive biomarkers is currently a major focus of investigation, in order to better identify those patients most likely to benefit from treatment. However, given that immunotherapies are designed to work directly with the patient’s own immune system rather than directly targeting the tumor, a different approach to identifying biomarkers to that which is typically used may be needed. Various approaches to identify potential biomarkers for immunotherapies are being evaluated, including expression of markers related to the target molecule or pathway (e.g. PD-L1 for therapies directed at the PD-1 pathway), treatment-emergent changes to the immune system or tumor microenvironment (e.g. absolute lymphocyte count, antibody responses to tumor antigens or change in TILs) and gene expression profiling of the patient or tumor (e.g. differential tumor expression of immune-related genes).

The unique modes of action of immunotherapeutic agents can also mean that they are associated with unique adverse event (AE) profiles as a result of immune system-related activity. These may differ from those seen with chemotherapy or targeted agents or, even if the same (e.g. pruritus, diarrhea), their etiology may differ. As such, different management strategies are required, including the use of specific algorithms for immune-related AEs, such as those developed for ipilimumab.

Another key area of research is the use of various immunotherapies in combination with one another and/or with chemotherapy, targeted agents or radiation. Several clinical studies have shown that combining different immunotherapies can improve outcomes and various combination approaches are being investigated.

The success of novel immunotherapies in melanoma and other cancers and their activity across multiple tumor types has led to an increased focus on their potential ability to improve outcomes across a range of solid and hematological cancers, including those for which current treatment options are limited and prognoses are typically poor. Recent developments in the use of immunotherapies in liver, kidney, and bladder cancers were discussed at the 2nd meeting of the Campania Society of Oncology Immunotherapy (SCITO) and are reported here.

### Hepatocellular carcinoma

Immune response is relevant to the development and growth of hepatocellular carcinoma (HCC), certain characteristics of which render it a potential target for immunotherapeutic intervention. There is an active recruitment of lymphocytes, which have specific mechanisms to recognize and bind to tumor endothelium and infiltrate tumor tissue, suggesting a potential for cytotoxic effector cell activation [7] and there is a correlation between the density of lymphocytic infiltrates in HCC lesions and prognosis [8]. In HCC patients after resection, low Tregs in combination with high intratumoral activated CD8+ cytotoxic cells was an independent prognostic factor for both improved disease-free survival (DFS) and OS [9]. In addition, programmed death-ligand 1 (PD-L1) overexpression has been shown to predict recurrence and survival in HCC patients after resection [10].

Immunotherapeutic strategies can be classified as either cell-based or non-cell-based. Potential cell-based approaches include dendritic cell vaccination, cytokine-induced killer cells, and NK cells or genetically modified T-cells, while non-cell based strategies include agents include cytokines (e.g. interferon-[IFN]-α), oncolytic viruses, peptide vaccines, and checkpoint inhibitor antibodies. Targeting CTLA-4, PD-1 and PD-L1 with monoclonal antibodies has revolutionized the field of cancer immunotherapy in recent years. Tremelimumab is a fully humanized IgG2 monoclonal antibody that antagonizes binding of CTLA-4 to B7 ligands which has shown antitumor activity in a variety of murine tumor models and in melanoma patients after single or multiple doses [11]. In an investigator-initiated, single-arm pilot phase II clinical trial conducted at three Spanish hospitals, tremelimumab showed antitumor and antiviral activity in HCC patients with chronic hepatitis C virus infection [12]. A total of 21 patients (mean age 63.5, 71 % male) with mostly advanced tumors (57 % Barcelona clinic liver cancer stage C) and varying degrees of liver dysfunction (43 % Child-Pugh class B) received tremelimumab at a dose of 15 mg/kg every 90 days for a maximum of four cycles or until tumor progression or severe toxicity. The main endpoint, tumor response, was evaluable in 17 patients; of these, three patients (18 %) had a partial response and 10 patients (59 %) had stable disease, which in three patients lasted for longer than 6 months. Four patients had progressive disease. Disease control rate was 76.4 %. In addition, a >50 % drop in the tumor marker α-fetoprotein (AFP) was observed in 36 % of patients with high baseline levels (>100 ng/ml). Median time to disease progression was 6.48 months (95 % confidence interval [CI]: 3.95–9.14), which compares favorably with results obtained with other targeted agents in HCC (Figure). Median OS was 8.2 months (95 % CI 4.6 − 21.3), with 12-month survival of 45 % and 18-month survival of 18 %. Tumor-specific responses were not tested but specific T-cell responses against hepatitis C virus antigens were observed in most patients. Tremelimumab was generally well tolerated with fatigue, hyporexia and skin rash (itching) being the most frequent AEs; most were grade 1−2. In another feasibility study, tremelimumab is being assessed in combination with transarterial chemoembolization (TACE) or radiofrequency ablation (RFA) in patients with HCC previously treated with sorafenib [13]. To date, 18 patients have been treated (TACE, n = 8; RFA, n = 10). The most frequent AE was pruritus and no dose-limiting toxicities have been reported. One patient developed pulmonitis and was taken off study but remained disease-free at 16 months. Anti-PD-1 and PD-L1 agents are also being investigated for their potential role in HCC, with a dose-escalation phase II clinical trial currently assessing the safety and efficacy of nivolumab in patients with advanced HCC with or without chronic viral hepatitis (NCT01658878).

Another potential approach is the development of peptide vaccines. Tumor-associated antigens are self-derived proteins rendered immunogenic in tumors by mutation or aberrant expression. In HCC patients, several tumor-associated antigens can spontaneously induce CD8+ T-cell responses including AFP, glypican-3 (GPC-3), and melanoma-associated gene-A1 (MAGE-A1). Single or multiple peptides derived from tumor-associated antigens may be used for cancer vaccination, usually in combination with an immunomodulatory adjuvant. This approach is being investigated by the EU-supported HEPAVAC project (www.hepavac.eu), the aim of which is to develop a universal vaccine comprising multiple tumor-associated peptides that are frequently and naturally presented on the surface of primary HCC cells. Up to 40 of such HLA class I and II restricted peptides will be selected in a multiepitope and multi-HLA allele strategy, and used for the induction of tumor-specific CD4+ T helper cell and cytotoxic CD8+ lymphocyte effector and memory immune responses in vaccinated patients. In the second stage, this will be complemented by an additional personalised vaccine (APVAC), for which mutated peptides will be identified from tumor lesions on a patient-specific basis and added to their respective drug compositions. As part of this initiative, a randomized, open-label, controlled, multicenter European phase I/II clinical trial will compare tumor resection/ablation alone with tumor resection/ablation plus vaccination.

Further opportunities for improving outcomes in patients with HCC may be provided by combined immunotherapy approaches, which have the potential to be more successful than single-agent interventions. Preclinical data suggest combinations of immunostimulatory monoclonal antibodies in conjunction with vaccines, adoptive T-cell therapy or conventional therapies (i.e. chemotherapy, targeted biological agents, radiation) are feasible and need to be clinically investigated. Moreover, evidence in mice with spontaneous HCC has shown proof of concept for curative synergistic combinations of an immunostimulatory antibody triplet combination (PD-L1 + CD137 + OX40) in conjunction with adoptive T-cell therapy, with extended survival in a CD8-dependent fashion [14].

### Renal cell cancer

The use of targeted agents, in particular vascular endothelial growth factor tyrosine kinase inhibitors (VEGF-TKIs), has resulted in improved response rates and progression-free survival (PFS) in patients with metastatic renal cell cancer (mRCC). However, most patients progress with maximum PFS generally less than 12 months. Patients with mRCC present with a variety of immune abnormalities, including cellular immune dysfunction, cytokine alterations and antigen presentation defects [15]. In addition, spontaneous regressions that could be explained by immune involvement have been observed in patients with mRCC, further suggesting it may be an immunogenic cancer type.

Treatment with IFN-α or interleukin-(IL)-2 has previously shown clinical activity in mRCC with durable responses being observed in some patients, although their use has been limited by severe toxicity [16]. A more promising novel immunotherapeutic strategy in mRCC is the development of vaccines based on autologous tumor cells, dendritic cells or peptides. IMA901 is a peptide vaccine consisting of 10 different tumor-associated peptides that has shown a clinical benefit in patients with mRCC. In a randomized phase II trial of 68 patients, those who produced an immune response to two or more of the tumor-associated peptides contained in IMA901 had significantly longer survival [17]. IMA901 is now being investigated in the ongoing multicenter phase III IMPRINT trial, which will assess whether it can prolong OS in patients with metastatic and/or locally advanced RCC when added to standard first-line therapy with sunitinib plus cyclophosphamide. Another vaccine in development is TroVax (modified vaccinia Ankara [MVA]-5T4), which consists of an attenuated pox virus vector expressing tumor-associated antigen 5T4. In a randomized, placebo-controlled phase III study of 733 patients with mRCC, treatment with TroVax in addition to standard of care was well tolerated but was not associated with any additional OS benefit when compared with standard of care alone [18]. However, the magnitude of the 5T4-specific antibody response induced by vaccination with MVA-5T4 was associated with enhanced patient survival while exploratory analyses suggested that a number of pre-treatment hematological factors could potentially be used to identify subsets of patients who might derive benefit (Figure). AGS-003 is an autologous dendritic cell vaccine prepared from fully matured and optimized monocyte-derived dendritic cells, which are co-electroporated with amplified tumor RNA plus synthetic CD40L RNA. In a phase II study, AGS-003 in combination with sunitinib was well tolerated and yielded supportive immunologic responses and prolonged survival in patients with mRCC [19]. AGS-003 in combination with sunitinib is currently being assessed in the pivotal randomized phase III ADAPT trial. In this study, patients in the experimental arm are being treated with a combination of AGS-003 and first-line sunitinib, while the comparator arm is receiving standard treatment g with sunitinib alone. After 6 weeks of sunitinib, patients will receive 8 doses of AGS-003 during the first year and, for those continuing to benefit, booster doses of AGS-003 will be given every 3 months thereafter in combination with standard targeted therapy. The primary endpoint of the trial is OS.

Anti-PD-1/PD-L1 agents may also have potential utility in patients with mRCC. In a phase I study, nivolumab had an objective response rate (ORR) of 29 % and a median duration of response (DOR) of 56 weeks. Median OS was not reached at 22 months and 1-year and 2-year OS rates were 70 % and 52 %, respectively [20]. In a phase II trial, 168 patients with clear-cell mRCC previously treated with VEGF inhibitors were randomly assigned to nivolumab 0.3, 2, or 10 mg/kg once every 3 weeks [21]. Respective ORRs were 20 %, 22 %, and 20 %. Median PFS was 2.7, 4.0, and 4.2 months, and median OS was 18.2, 25.5 and 24.7 months, respectively. The most common treatment-related AE was fatigue (24 %, 22 %, and 35 %, respectively). Nineteen patients (11 %) experienced grade 3–4 treatment-related AEs. In a prospective biomarker-based study, nivolumab showed clinical activity in patients with previously treated and untreated mRCC [22]. CD3+ and CD8 + T-cell infiltrates increased by a median of 70 % and 88 % respectively from baseline to day 8 of the second cycle. Although responses were numerically higher in PD-L1-positive patients, they were also seen in PD-L1-negative patients. Changes in biomarkers were consistent with PD-1 inhibition and provided evidence of immunomodulatory effects in serum and in the tumor microenvironment.

As with other tumor types, combined immunotherapy may offer improved outcomes in mRCC. A combined CTLA-4 blockade and PD-1 blockade approach was assessed in the phase I CA209016 study in which patients with mRCC received nivolumab 3 mg/kg plus ipilimumab 1 mg/kg (N3 + I1) (n = 21) or nivolumab 1 mg/kg plus ipilimumab 3 mg/kg (N1 + I3) (n = 23) every 3 weeks for 4 doses followed by nivolumab 3 mg/kg every 2 weeks until disease progression or unacceptable toxicity [23]. Treatment-related AEs were seen in 89 % of patients with grade 3–4 AEs occurring in 43 % of patients. The most frequent were increased lipase, increased ALT, diarrhea, colitis, and increased amylase. Overall, the safety profile was acceptable with only seven patients (N3 + I1: 2; N1 + I3: 5) discontinued due to any-grade related AE. Confirmed ORR was 43 % (N3 + I1) and 48 % (N1 + I3), while median DOR was 31.1 weeks in the N3 + I1 arm and was not reached in the N1 + I3 arm. PFS at 24 weeks was 65 % with N3 + I1 and 64 % with N1 + I3.

Another option is combining PD-1 blockade with VEGFR-TKIs, which may increase the antitumor efficacy of PD-1 blockade by reducing tumor-infiltrating Tregs and enhancing the activity of cytotoxic T cells. In the CA209-016 phase I study, mRCC patients received nivolumab in combination with sunitinib 50 mg (4 weeks on, 2 weeks off) or pazopanib 800 mg daily, until progression or unacceptable toxicity [24]. Confirmed ORR was 52 % with sunitinib plus nivolumab and 45 % with pazopanib plus nivolumab, while median DOR was 37.1 and 30.1 weeks. PFS at 24 weeks was 78 %, which is comparable to sunitinib alone in the first-line treatment of metastatic RCC. However, dose-limiting liver toxicity in the pazopanib arm led to its closure. In these studies of nivolumab with ipilimumab, sunitinib, or pazopanib, PD-1-positive tumor status did not appear to predict a response, with significant proportions of PD-L1 negative patients also responding to treatment. Several other planned or ongoing trials are also exploring other anti-PD-1 combination approaches, including with bevacizumab.

### Bladder cancer

Metastatic urothelial bladder cancer (UBC) is associated with limited treatment options and a poor prognosis. Response rates with salvage chemotherapy for advanced UBC continue to be dismal, generally around 20 % or less, with median OS typically in the region of 6–9 months. UBC is associated with high mutational complexity, with similar rates of mutation as observed in patients exposed to tobacco or other environmental carcinogens, and there is already a precedent for the use of immune therapy in UBC, with intravesicular BCG the standard of care for non-muscle invasive bladder carcinoma.

Urothelial bladder tumors have been shown to have levels of PD-1 expression similar to other solid tumors, with PD-1 expression noted in 45 % of 83 muscle invasive bladder cancers (Table) [25]. Atezolizumab (MPDL3280A) is an engineered anti-PD-L1 antibody that inhibits the binding of PD-L1 to PD-1 and B7.1. In patients with non-selected solid tumors, including NSCLC, RCC, melanoma, colorectal cancer and gastric cancer, atezolizumab had an ORR of 21 % (25/122), with several patients demonstrating tumor shrinkage within days of initiating treatment [26]. In a phase I expansion study, 85 previously-treated patients with metastatic UBC received atezolizumab and were evaluable for efficacy [27]. Of these, 46 were PD-L1-positive and 38 were PD-L1-negative, with one having unknown PD-L1 status. In PD-L1-positive patients, ORR was 46 % (95 % CI: 31−61 %) compared with 16 % (95 % CI: 6 − 31 %) in PD-L1-negative patients, with median response durations not yet reached. Median PFS was 24 weeks (95 % CI: 12 − not reached [NR]) for PD-L1-positive patients and 8 weeks (95 % CI: 6−12) for PD-L1-negative patients. OS rates at 24 weeks were 85 % (95 % CI: 74−96 %) and 71 % (95 % CI: 54−88 %) for PD-L1-positive or negative patients, respectively, with medians not yet reached. Drug-related AEs occurred in 64 % of 87 safety-evaluable patients (mostly fatigue, asthenia or nausea) and 8 % had a grade 3−4 drug-related AE. Twelve-percent of patients had an immune-related AE. No treatment-related deaths were observed. Increases in circulating IFN-γ, IL-18 and activated CD8+ T-cells were observed following treatment. On-treatment plasma tumor burden markers, but not baseline markers, were associated with response. Atezolizumab is now being assessed in patients with UBC in phase II (NCT02108652) and phase III trials (NCT02302807).

Other anti-PD-1 agents are also being assessed for bladder cancer. The ongoing KEYNOTE-012 study of pembrolizumab in PD-L1-positive patients with advanced solid tumors included a cohort of 33 patients with recurrent or metastatic UBC [28]. ORR was 24 % (95 % CI: 10−44 %), with three (10 %) complete and four (14 %) partial responses. Target lesions were reduced in 64 % of patients. Six responses were ongoing at a median duration of follow-up of 11 months and response duration was 16–40+ weeks (median not reached). Median PFS was 8.6 weeks (95 % CI: 7.4−14.1) and median OS was 9.3 months (95 % CI: 3.6–NR) with an OS rate at 6 months of 58.0 %. Treatment was generally well tolerated, with only one patient discontinuation due to a treatment-related AE. A randomized phase III study of pembrolizumab in advanced UBC is planned (KEYNOTE-045). Nivolumab is also being investigated as a treatment for metastatic or unresectable UBC (NCT02387996).

## Conclusions

Immunotherapies offer the potential for improving outcomes in patients with cancer through immune adaptability and memory, while targeting the immune system rather than the tumor offers the potential for activity across multiple cancer types irrespective of mutational status or tumor histology. Their various unique mechanisms of action and safety profiles offer the opportunity for combination therapy strategies, either with one another and/or together with other treatment modalities. Such combination approaches have already begun to show promising results and look likely to offer additional clinical benefit compared with single-agent therapies. Although the research focus and main advances have to date been largely in melanoma, immunotherapies are being actively investigated in many other cancer types, including those where treatment options for patients are limited. Currently ongoing and planned studies should help bring the benefits of these novel immunotherapies within the reach of patients with a wide range of cancers.

## Abbreviations

AE: Adverse event
AFP: α-fetoprotein
APVAC: Additional personalised vaccine
BCG: Bacillus Calmette-Guérin
CD: Cluster of differentiation
CI: Confidence interval
CTLA-4: Cytotoxic T-lymphocyte-associated protein 4
DFS: Disease-free survival
DOR: Duration of response
EU: European Union
GPC-3: Glypican-3
HCC: Hepatocellular carcinoma
HLA: Human leukocyte antigen
IFN: Interferon
IL: Interleukin
MAGE-A1: Melanoma-associated gene-A1
MRCC: Metastatic renal cell cancer
NK: Natural killer
NSCLC: Non-small-cell lung cancer
ORR: Objective response rate
OS: Overall survival
PD-1: Programmed death protein 1
PD-L1: Programmed death-ligand 1
PFS: Progression-free survival
RECIST: Response Evaluation Criteria in Solid Tumors
RFA: Radiofrequency ablation
RNA: Ribonucleic acid
SCITO: Campania Society of Oncology Immunotherapy
TACE: Transarterial chemoembolization
TIL: Tumor-infiltrating lymphocyte
TKI: Tyrosine kinase inhibitor
Tregs: T-regulatory cells
UBC: Urothelial bladder cancer
VEGF: Vascular endothelial growth factor
WHO: World Health Organization
